# Chaotic and complex dynamics expose the limits of counterfactual reasoning

**DOI:** 10.1038/s41598-026-52349-2

**Published:** 2026-06-02

**Authors:** Yahya Aalaila, Gerrit Großmann, Sumantrak Mukherjee, Jonas Wahl, Sebastian Vollmer

**Affiliations:** 1 Data Science and its Applications Research Group, German Research Center for Artificial Intelligence (DFKI), Kaiserslautern, Germany; 2 Research Department Neuro-Mechanistic Modeling, German Research Center for Artificial Intelligence (DFKI), Saarbrücken, Germany; 3 UM6P College of Computing, Mohammed VI Polytechnic University, Benguerir, Morocco; 4 Department of Philosophy, University of Bergen, Sydnesplassen 12–13, 5007 Bergen, Norway; 5 Department of Computer Science, Rhineland Palatinate Technical University of Kaiserslautern-Landau (RPTU), Rhineland-Palatinate 67663 Kaiserslautern, Germany

**Keywords:** Engineering, Mathematics and computing

## Abstract

Counterfactual reasoning, a cornerstone of human cognition and decision-making, is often seen as the “holy grail” of causal learning, with applications ranging from interpreting machine learning models to promoting algorithmic fairness. While counterfactual reasoning has been extensively studied in contexts with clearly defined static causal models, many real-world scenarios reside in dynamic settings often involving model and parameter uncertainty, observational noise, and chaotic behavior. The reliability of counterfactual analysis in such settings remains largely unexplored. In this work, we investigate the limitations of counterfactual reasoning in dynamic settings. We specifically focus on counterfactual sequence estimation and demonstrate empirically that even modest levels of model uncertainty or observational noise can lead to dramatic deviations between predicted and true counterfactual trajectories. Our findings urge caution when applying counterfactual reasoning in dynamical systems, particularly those that may exhibit complex, chaotic behavior, and highlight fundamental limitations in answering certain counterfactual queries reliably.

## Introduction

Imagine that a student, Alice, is thrilled to have passed her final exam. She wonders what if she hadn’t joined that study group last month? Would she still have succeeded? What if she had chosen an easier path than taking this challenging course? Would she have also completed her studies at a different university? Imagining such hypothetical realities to reason about *“What if”* questions is a cornerstone of human cognition^1^. Investigating this type of reflection, known as counterfactual reasoning, has a long history in philosophy^2,3^, and psychology^4–6^. For example, in^5^, participants were asked to make causal judgments about collisions between moving balls. In the experiment, they watched short video clips where two balls collided and one ball either passed through a gate, missed it, or nearly passed through. Using their intuitive understanding of physics, participants mentally simulated what would have happened if the candidate cause had been removed from the scene, effectively playing out the counterfactual trajectory of ball B had the collision not occurred^7^. Counterfactual reasoning is not limited to simple, controlled tasks, but also scales to socially and politically complex systems where uncertainty is high. The work^8^ shows that people make counterfactual causal judgments even in a highly nonlinear, multivariate context such as the 2020 U.S. presidential election. Participants judged the causal contribution of individual states to the overall outcome, and their attributions closely matched the computational models based on counterfactual effect sizes across simulated election outcomes. More recent studies have shown that people strategically manage chaos when reasoning counterfactually in dynamic systems. For instance, prior work in cognitive psychology highlights that humans rely on mental simulations which are continually updated in light of observed input, ensuring that their internal representations remain aligned with reality rather than drifting indefinitely^9^.

In Pearl’s causal framework, counterfactual reasoning represents the third and highest level of the ladder of causality^10^. Counterfactual reasoning is not only used by individuals in thought experiments, but is also common in science, which remains controversial, as counterfactual claims are typically not falsifiable^11^. For instance, in Rubin’s *potential outcome framework*^12^, the effect of a (e.g. medical) treatment is estimated by comparing the treated population to a counterfactual untreated one. More concretely^13^, used a counterfactual study to estimate the number of deaths related to Covid-19 assuming different transmission rates and^14^ studied how (hypothetical) social support of a patient would have changed their mental health outcomes. Investigate^15^ and^16^ the outcomes of hypothetical treatments to a patient, while^17^ explore counterfactual trajectories in physical systems.

The framework of Structural Causal Models (SCMs) offers a robust framework for the formalization of causality and provides a general recipe for computing counterfactuals based on three steps^10^: abduction, action, and prediction. While this is widely accepted as an elegant and principled way of formalizing *“What if”* questions, this method requires perfect world knowledge (i.e., a known SCM) and absence of measurement noise. In practice, SCMs are a coarse abstraction of the real world, and only noisy observations are recorded. Consequently, even when we understand the laws governing a dynamical system, we rely on numerical methods to approximate unobserved parameters and variables. This challenge is further complicated by the fact that the systems with which we interact in the real world are often highly complex. Moreover, many natural and engineered systems, while deterministic, exhibit chaotic behavior so that small differences in initial conditions can lead to vastly different outcomes, a phenomenon known as sensitive dependence on initial conditions. This property was first highlighted in Lorenz’s pioneering work on atmospheric convection, which provided one of the earliest demonstrations of deterministic unpredictability^18^. Since then, chaotic systems like Lorenz and Rössler have become canonical testbeds for studying sensitive dependence on initial conditions^18,19^. More recent studies, however, have extended this perspective to multi-stable and complex chaotic systems, where multiple attractors coexist and the long-term state of the system depends sensitively on the basin of initial conditions, and small perturbations can trigger abrupt transitions between these attractors^20–22^.

Conceptually, in purely chaotic systems, counterfactual reasoning becomes impossible in principle. Because any arbitrarily small perturbation of the initial state leads to exponential divergence, counterfactual interventions to any variable would likely cause the system to evolve along an entirely different trajectory. In such extreme regimes, historical “what if” thought experiments effectively collapse into invariant distributions of the system, akin to the classical “butterfly effect” analogies^23,24^. However, when modeling real-life phenomena in a dynamic setting with hidden states, noise, partial observations, and uncertainty, it becomes exceedingly difficult to determine whether the underlying system exhibits chaotic behavior or simply involves complex nonlinear behavior. In fact, there is an ongoing debate about the identifiability of chaos in real world contexts. For example, the work in^25^ shows how biological feedback loops can appear chaotic, yet conclusive evidence for any specific system remains unattainable. Similarly,^11^ examines how cardiac processes may exhibit chaotic dynamics, while^26^ demonstrates that even small gene regulatory networks can generate complex oscillations. Along the same lines,^27^ showed how cortical microcircuits exposed to NMDAR antibodies can be pushed into a critical regime, where small parameter perturbations trigger phase-transition–like changes in network dynamics. Although not all of these works explicitly focus on chaos, they underscore that subtle feedback loops in biological systems might yield chaotic-like effects. As a result, when we attempt to model real-life phenomena, it is difficult to ascertain whether they are truly chaotic or highly complex–an uncertainty that carries serious implications for counterfactual reasoning. If even a slight parameter or measurement error can spur major deviations in a chaotic regime, then counterfactual predictions in such settings must be interpreted with caution.

In light of these debates on whether real-world systems may exhibit chaotic or complex dynamics akin to chaotic behavior, our work examines what happens if we treat them as though they do, even under strong, seemingly generous assumptions. Specifically, we explore the case where a system truly exhibits sensitive dependence on initial conditions, as exemplified by classical chaotic systems such as the Lorenz and Rössler models, alongside process noise, observational noise, and parameter uncertainty. By combining a state-space model (to capture low-level ODE-based evolution) with a structural causal framework (to reason about hypothetical interventions), we show that, even with full knowledge of the governing equations, small initial perturbations or slight parameter misestimates may still lead to vastly different trajectories. By comparing counterfactual trajectories when parameters are (1) perfectly known, (2) slightly misestimated, and (3) drawn from a posterior distribution, we show that while factual state estimation can remain accurate, counterfactual predictions can become severely unreliable. This highlights how, even with full knowledge of an ODE’s form and a sophisticated inference method, the mere possibility of chaotic behavior coupled with noisy observations and slight uncertainty can pose a fundamental obstacle for reliable counterfactual reasoning in real-world settings.

**Contributions**

- To the best of our knowledge, this work is the first to demonstrate how to perform counterfactual reasoning in the context of dynamical systems without assuming (1) perfect observations of the system (i.e., no noise in the process or observations) and without (2) perfect knowledge of the underlying dynamical laws. To this end, we provide a conceptualization and mathematical formalization of the relationship between dynamical systems with inherent stochasticity and Structural Causal Models.
- Unlike previous work, we consider the computation of counterfactual predictions in chaotic systems with parameter uncertainty. We show that the reliability of counterfactual predictions cannot be derived (in a trivial way) from observing the original system.

The manuscript is organized as follows: We lay out our notation and formalize dynamical models and (dynamic) SCMs in section “Background”. Our central method is presented in Section “Methodology”, where we show how to compute counterfactuals in realistic scenarios using Bayesian filtering. Section “Results” uses numerical experiments to illustrate the limitations of counterfactual reasoning in the context of noise, uncertainty, chaos, and abstraction. A discussion of related works in section “Related work” provides context for our contributions. Finally, the discussion, conclusion and future directions complete the manuscript.

## Related work

The term “counterfactual” has been used in various contexts, particularly in *Counterfactual Explanations *^28^ to interpret the behavior of models by describing how changes in inputs would lead to different outputs, answering queries such as *“Had this feature been different, the model would have predicted x instead of y”*. *Counterfactual Regret Minimization* is used for game strategies by considering alternative actions that could have been taken to minimize regret and improve outcomes ^29^. *Counterfactual Fairness *^30^ is a concept that reflects fairness in AI, stipulating that the model’s prediction should be the same in both the observed and counterfactual scenarios where only sensitive attributes are altered.

While counterfactuals are ubiquitous in human reasoning, the reliability of counterfactual reasoning is often questioned. Prior work ^31–33^ and^34^ underscores the risks of model dependence and post-hoc interpretability, where counterfactual explanations may be based on model artifacts rather than real data, potentially misleading users. Prior work ^35^ stresses that counterfactual inferences often rely on speculative assumptions that lack empirical backing, making verification essential. They recommend methods to detect and control model dependence, particularly for extreme counterfactuals where the assumptions are most fragile. Recent work ^36^ highlights the instability of counterfactual predictions under perturbations, calling into question the reliability of counterfactual predictions, especially for real-world applications. Complementary work ^37^ identifies key evaluation gaps in counterfactual explanations for XAI, noting the need for better validation, standardized benchmarks, and practical feasibility assessments to advance the field. In addition, the work in^38^ has examined counterfactual generation in the context of spatiotemporal point processes where it shows counterintuitive behavior with different, statistically equivalent, simulation methods. A parallel line of work has studied counterfactual reasoning in human psychology. A study in cognitive psychology ^39^ explored how counterfactual thinking can make people’s judgments more extreme, and how human biases and previous experiences play a key role in people’s counterfactual judgments.

While counterfactual thinking has been used in different contexts, we are particularly interested in counterfactuals in the context of dynamical systems. Studies ^40–44 focus^ on counterfactual learning of physical dynamics in mechanical systems, which allows predicting the outcomes of physical processes under counterfactual scenarios. The work in ^40^ introduces CoPhy, a framework that learns physically interpretable models by training on hypothetical interventions, allowing it to predict how a scene might evolve under altered initial conditions or forces. The study in ^41 builds^ on this idea with Filtered-CoPhy, extending counterfactual reasoning to pixel-level reconstructions and emphasizing unsupervised learning of alternative physical trajectories. The approach ^42^ proposes CLEVReR, a benchmark specifically designed to test a model’s capacity for causal and counterfactual reasoning in collision-based scenarios, thereby assessing whether models can correctly infer “what would happen if” after object interactions deviate from the observed course. Meanwhile, the work in ^43^ develops a dynamic visual reasoning method that integrates visual inputs and language cues to learn differentiable physics models, enhancing the model’s ability to generate counterfactual outcomes for complex mechanical events. However, these approaches are typically confined to simulated or visual domains with clean object-level structure and stable Newtonian dynamics, and thus do not directly engage with the instability of counterfactuals in noisy, continuous-time dynamical systems. Beyond this, related work in neuroscience has employed Dynamic Causal Modeling (DCM) to infer hidden states and parameters in continuous-time systems, with applications ranging from consistent spectral estimation ^45^ to psychiatric disorders and microcircuit perturbations ^27^. From a complementary angle, cognitive psychology has examined how humans perform counterfactual reasoning in dynamic environments, including collision-based judgments ^5,7^ and causal attributions in complex political contexts such as the 2020 U.S. election ^8^.

Recent studies have approached counterfactual reasoning in dynamical systems by constraining the underlying causal model with explicit structural assumptions. For instance, the work in ^46^ fixes a single structural causal model (SCM), assuming a predetermined causal mechanism for the environment. Counterfactual analyses are then carried out within this fixed latent-state dynamical model, which enables tractable inference but does not account for uncertainty over possible causal mechanisms ^28,47^ also adopt strong modeling constraints by employing the Gumbel-max SCM, which enforces counterfactual stability by ensuring predictions remain invariant to small perturbations. While this guarantees unique and well-defined counterfactual outcomes, it narrows the modeling space and precludes exploration of alternative causal structures that may also be consistent with the observational data. Recognizing this limitation, the studies in ^48,49^ propose a robust framework for bounding counterfactual outcomes in dynamic latent-state models, with significant applications in sensitive areas such as healthcare and insurance claims. Given that counterfactual queries in partially observed systems are inherently non-identifiable, they instead focus on deriving provable upper and lower bounds rather than attempting to recover exact outcomes. Their approach integrates particle-filtering methods to estimate hidden states from observed sequences, followed by a polynomial optimization procedure constrained by known marginal distributions and domain-specific assumptions, such as path-wise monotonicity and structural stability. Implicit in this framework, however, is the assumption that the underlying system dynamics remain sufficiently stable and predictable. In systems exhibiting extreme complexity or sensitive dependence on initial conditions–hallmarks of chaotic behavior–small errors in state estimation or unrecognized parameter uncertainties can amplify dramatically, rendering the resulting bounds overly optimistic or misleadingly narrow. Such limitations are particularly consequential in medical contexts, where subtle dynamic instabilities may critically affect patient outcomes.

## Background

This section introduces three core concepts and their notation: dynamical systems, represented through Ordinary Differential Equations (ODEs) and State-Space Models (SSMs); inference, applying Sequential Monte Carlo (SMC) methods to account for uncertainty and noise; and Structural Causal Models (SCMs), which formalize causality within these systems. We also outline the process of translating ODEs into SSMs, and further, into SCMs, enabling us to apply causal frameworks to well-established dynamical systems from the literature.

### Dynamical systems

#### Ordinary differential equations

Ordinary Differential Equations (ODEs) provide a universal language to describe deterministic systems via equations that determine how variables change in time as a function of other variables^50^. Consider time-indexed state variables $X_{i}(t) \in \mathbb {R}$, for $i = 1, \dots , d$. We denote $\textbf{X}(t) = \left( X_1(t), X_2(t), \dots , X_d(t)\right)$ the vector of all $X_i(t)$. An ODE is defined by:

1 $$\begin{aligned} \frac{d}{dt} \textbf{X}(t) = h_{\boldsymbol{\theta }}(\textbf{X}(t)), \quad \textbf{X}(0) = \textbf{X}_0 \in \mathbb {R}^{d}. \end{aligned}$$

where $\textbf{h}_{\boldsymbol{\theta }} = \left( h_{1, \boldsymbol{\theta }},\dots ,h_{d, \boldsymbol{\theta }}\right)$ applies $h_{i, \boldsymbol{\theta }}$ on each component $X_i(t)$, specifying how each state component evolves over time. The function $\textbf{h}_{\boldsymbol{\theta }}$ depends on parameters $\boldsymbol{\theta } = \left( \theta _1, \dots , \theta _p \right)$ that govern the evolution of the system. For each $\boldsymbol{\theta } \in \mathbb {R}^{p}$ and initial condition $\textbf{X}_0\in \mathbb {R}^{d}$ Eq. (1) describes a unique system.

While ODEs describe the continuous evolution of a system’s state over time as $\textbf{X}(t)$, numerical simulations often require discretizing the system to make it tractable for computational methods. To approximate the continuous behavior, we introduce a time step $\Delta$ and consider a set of discrete time points $t_i = i \cdot \Delta$, approximating the state of the system at these points as $\textbf{X}_{t_i}$ at the discrete time $t_i$. For ease of notation, we will refer to the system state using the notation $\textbf{X}_{t}$ to represent the state at discrete time step *t*.

#### State-space models

State-space models (SSMs) extend systems described by ODEs by modeling dynamical systems where the true state is hidden and only noisy observations are available at each discrete time point. These models consist of two main components: the *state equation*, which describes the evolution of the hidden state $\textbf{X}_t \in \mathbb {R}^{d}$, and the *observation equation*, which links the hidden state to the observed data $\textbf{Y}_t\in \mathbb {R}^{d}$^51^ (w.l.o.g., we assume the same dimension). The hidden states are assumed to follow a Markov process, and the observations depend only on the state at the corresponding time *t*. Both the hidden states and the observations are subject to additive Gaussian noise. The evolution of $\textbf{X}_t$ is described by the following equations:

2 $$\begin{aligned} \textbf{X}_t&= F(\textbf{X}_{t-1}, \boldsymbol{\theta }) + \textbf{U}_t \end{aligned}$$

3 $$\begin{aligned} \textbf{Y}_t&= \textbf{H} \textbf{X}_t + \textbf{W}_t, \end{aligned}$$

Here, $F(\textbf{X}_{t-1}, \boldsymbol{\theta })$ is the *forward operator*, representing the discretized deterministic part of the system’s evolution. The process noise $\textbf{U}_t$ and the observation noise $\textbf{W}_t$ are considered to be normally distributed with mean $\textbf{0} \in \mathbb {R}^d$ and variance $\textbf{R}\in \mathbb {R}^{d\times d}$ and $\textbf{Q}\in \mathbb {R}^{d\times d}$, respectively. The matrix $\textbf{H} \in \mathbb {R}^{d \times d}$ is the observation model that linearly maps the hidden state space to the observation space. It defines how each component of the hidden state $\textbf{X}_t$ contributes to the observed data $\textbf{Y}_t$.

### Sequential Monte Carlo methods for state and parameter estimation

Estimating hidden states and unknown parameters in dynamical systems from noisy observations is a fundamental challenge in many scientific and engineering applications. Sequential Monte Carlo (SMC) methods, commonly known as particle filters, offer a powerful framework for tackling this problem, especially in the context of nonlinear and non-Gaussian state-space models. In principle, there are two main approaches to joint state-parameter estimation in the sequential framework^52^: (1) treating the parameters as slowly varying hidden states, or (2) updating parameters after filtering a batch of observations. In this work, we align with the latter, and employ the *nested particle filter* approach introduced by^53^ to jointly estimate the hidden states $\textbf{X}_t$ and the system parameters $\boldsymbol{\theta }$. The NPF extends traditional particle filtering by incorporating an additional layer dedicated to parameter estimation. The algorithm consists of two nested layers of particle filters: an *outer* filter that approximates the parameters posterior $\boldsymbol{\theta }$ given the observations and a set of *inner* filters, one per sample generated in the outer filter, that yields approximations of the hidden state posterior that result for $\textbf{X}_t$ conditional on the observations and each specific particle of $\boldsymbol{\theta }$. This approach can be broken down to the following steps:

- Generate *M* parameter particles $\{\boldsymbol{\theta }^{(m)}\}_{1\le m \le M}$ from the prior distribution $\pi _0$ and $M\times N$ hidden states particles $\{\textbf{x}_t^{(n,m)}\}_{1\le n\le N}^{1\le m\le M}$ from the prior distribution $\tau _0$.
- At each time step *t*, the state particles $\{\textbf{x}_t^{(n,m)}\}$ are propagated using the system dynamics, and their weights are updated based on how they match the observations.
- The parameter particles $\{\boldsymbol{\theta }^{(m)}\}$ are then updated by aggregating the information from the inner filter (state particles) and computing new weights based on how well the states explain the observations.

Nested filtering approaches track the joint posterior $(\textbf{X}_t, \boldsymbol{\theta })$ using only observations up till time *t*. In our setting, however, we require smoothing because counterfactual inference must condition on the entire sequence. Following the abduct-action-formulation (outlined in the next section), the backward pass incorporates the abducted exogenous noise consistently across all time steps. Concretely, we apply a backward smoothing algorithm as detailed in^54^. This procedure adjusts the particle weights $w_t^{(n,m)}$ post hoc to account for the entire observation sequence, producing smoothed weights $\tilde{w}_t^{(n,m)}$. Based on these smoothed weights, we obtain refined estimates of the states and parameters.

### Structural causal models

A Structural Causal Model (SCM) describes a deterministic transformation from a set of exogenous (noise) variables to a set of endogenous (system) variables through a specific data-generating process. One of its core components is a directed acyclic graph (DAG) that represents the causal relationships between variables and can be used to answer causal queries. We define an SCM as a tuple $(\textbf{U}, \textbf{V}, \textbf{f}, P(\textbf{U}), \textbf{PA})$, where: $\textbf{U} = \{U_1, \dots , U_m\}$ and $\textbf{V} = \{V_{1}, \dots , V_n\}$ represent the sets of exogenous (noise) variables and endogenous (system) variables, respectively. The set of parent-child relationships, $\textbf{PA} = \{PA_1, \dots , PA_n\}$, defines each, $V_i \in \textbf{V}$ with a corresponding set of parent variables $PA_i \subseteq \textbf{V} \cup \textbf{U}$ while $U_i \in \textbf{U}$ are root nodes. We assume an acyclic directed graph structure, allowing for endogenous variables without parents and those with multiple exogenous parents. The set of structural functions $\textbf{f} = \{f_1(\cdot ), \dots , f_n(\cdot )\}$ describes how each $V_i$ is generated by its causal parents, with $V_i := f_i(\text {PA}_i)$. The exogenous variables follow a probability distribution $P(\textbf{U})$, typically assuming independence between noise variables.

#### Computing counterfactuals

SCMs make it possible to study the effects of modifications to the data generating process (e.g., by fixing the value of a specific $V_i$ or removing a dependency) and “imagining” how the output might look like. Specifically, we need to take an SCM and an observation as input, and hypothesize about how the observational data would have looked like under a different (modified) SCM. Computing counterfactuals involves a three-step procedure^10^: **Abduction**, where exogenous variables are inferred from observed data, computing the posterior distribution of noise variables; **Action**, which modifies the SCM by fixing certain variables or relationships; and **Prediction**, solving the modified SCM using the inferred noise posterior rather than the original distribution.

The abduction step is computationally the most intensive, as it requires solving an inverse problem. While we defined an SCM as a transformation from noise variables to endogenous variables, the abduction step reverses this process by identifying the noise variables that lead to a specific assignment of endogenous variables. In Bayesian terms, the observations update the prior distribution $P(\textbf{U})$ to a posterior distribution conditioned on the evidence. Typically, this posterior distribution is more complex than the prior, as it lacks a closed analytical form and loses the independence between noise variables.

The intuitive reasoning behind this approach is as follows: We assume that all inherent stochastic and environmental influences–conceptually considered as noise–are encapsulated within the noise variables of the SCM. By fixing the noise (as accurately as possible), we essentially stabilize all environmental effects and other disturbances, allowing the system to be re-evaluated under specific modifications. The underlying assumption of this process is that sufficient knowledge about the noise can be captured through observations and that the noise variables in the modified SCM correspond meaningfully to those in the original model.

#### Dynamical SCMs

SCMs are typically acyclic and primarily used to model static systems. However, generalizing SCMs to dynamical systems presents multiple possibilities. The first arises from state–space modeling traditions in neuroscience and related fields^45,55–57^. In this setting, DCMs are formulated as continuous-time dynamical systems in which hidden states and parameters are inferred jointly, typically using variational Bayesian or spectral methods. This approach has been extensively applied to modeling effective connectivity in the brain^55^, and more recently to modelling trajectories of psychiatric disorders^56^, as well as epidemiological forecasting^57^. Unlike SCMs, DCMs are not restricted to acyclic graphs, since temporal unfolding naturally introduces cycles and feedback loops. However, these limitations can be finessed by considering dynamic and temporal precedence within structural causal modeling. This is because the arrow of time turns directed cyclic graphs into directed acyclic graphs, when the nodes are deployed over successive time points^55^. Alternatively,^58^ introduced dynamical SCMs, which are similar but incorporate additional parametric assumptions about how the unfolded SCM evolves over time. However, to avoid unnecessary assumptions and design choices, we adopt the simpler unfolding method. This approach is principled and aligns well with the SSM framework, making it suitable for modeling dynamical systems over discrete time steps.

## Methodology

In this section, we outline how we model the dynamical system as an SSM to infer the system’s parameters and hidden states. Importantly, we establish how SSMs can be interpreted as an SCM unfolded over time, shifting the focus from merely describing system dynamics to the underlying causal relationships between variables and external influences. While the SSM provides a low-level, quantitative description of the system’s evolution, the SCM framework offers a higher-level perspective that captures how changes in variables or external conditions propagate through the system. This perspective allows us to compute counterfactuals by first performing an abduction step, where we infer the latent variables and noise terms from observations. The subsequent prediction step depends on the type of counterfactual intervention being considered, such as perturbing initial conditions.

### Translating SSMs to SCMs

Converting an SSM into an SCM is simple by treating all $\textbf{X}_t$ and $\textbf{Y}_t$ as endogenous variables, while all $\textbf{U}$ and $\textbf{W}$ are considered noise variables. The hidden state $\textbf{X}_t$ depends on the previous state $\textbf{X}_{t-1}$ (with $t=0$ as user-specified) and the process noise. Likewise, the observation $\textbf{Y}_t$ is determined by $\textbf{X}_t$ and the observational noise. Thus, the state equation (2) $\textbf{X}_{t} = F(\textbf{X}_{t-1}, \boldsymbol{\theta }) + \textbf{U}_t$ and observation equation (3) $\textbf{Y}_t = \textbf{H} \textbf{X}_t + \textbf{W}_t$ are considered as the structural equation from the SCM perspective. Handling the parameter $\boldsymbol{\theta }$ is less straightforward. In principle, it can be hard-coded into the structural functions of the SCM. However, since the system parameters are typically unknown and need to be inferred, a more principled approach is to represent them as nodes in the SCM.

**Fig. 1:**
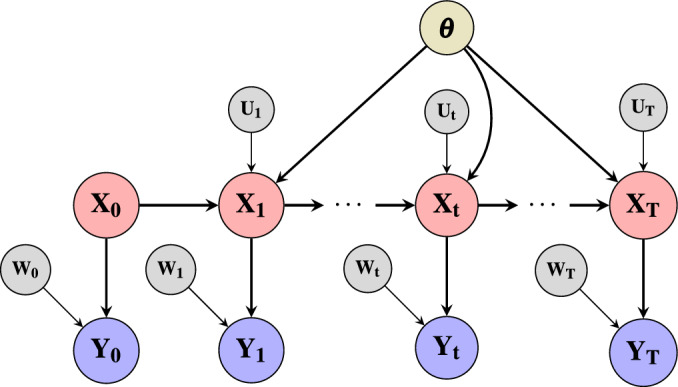
Graphical representation that highlights the equivalence between SSMs and SCMs.

Figure 1 provides a causal graph illustrating the evolution of the hidden states under the influence of process noise. Each node represents a causal relationship at time *t*, demonstrating how hidden states evolve sequentially and influence the observed outputs. This causal interpretation of SSMs allows us to leverage the SCM framework for reasoning about interventions and counterfactual scenarios, where each time step becomes a causal node governed by the previous state and the noise perturbations. Having established this interpretation, we now proceed to describe how counterfactual trajectories are generated.

### Estimating the noise posterior with NPF smoothing

It is virtually infeasible to provide a closed-form expression for the joint posterior distribution of the hidden state and parameters, but we can jointly estimate the hidden states and system parameters using nested particle filter-smoothing approaches. To this end, we follow the conventional NPF approach detailed in^53^.

### Initialization

- **Parameter particles:** Draw $M$ samples $\{\boldsymbol{\theta }^{(m)}\}_{m=1}^{M}$ from the parameter prior $\pi _0$. This captures our initial uncertainty about $\theta$.
- **State particles:** For each parameter $\boldsymbol{\theta }^{(m)}$, generate $N$ state particles $\{\textbf{x}_0^{(n,m)}\}_{n=1}^{N}$ from the state prior. In our work, the state evolution is modeled by the forward operator $F$ defined above.

### Recursive update

1. **Parameter Jittering (Mutation):** For each parameter particle $\boldsymbol{\theta }^{(m)}_{t-1}$, apply a jittering kernel $\kappa _N$ to obtain a new candidate
  $$\bar{\boldsymbol{\theta }}^{(m)}_t \sim \kappa _N(\cdot \mid \boldsymbol{\theta }^{(m)}_{t-1}).$$
2. **State propagation:** For each jittered parameter $\bar{\boldsymbol{\theta }}^{(m)}_t$ and its associated state particles $\{\textbf{x}_{t-1}^{(n,m)}\}_{n=1}^{N}$, update the state by
  $$\textbf{x}_{t}^{(n,m)} = F_t(\textbf{x}_{t-1}^{(n,m)}, \boldsymbol{\theta }^{(m)}) + \textbf{U}_t, \quad \textbf{U}_t \sim \mathcal {N}(\textbf{0}, \textbf{R}), \; 1\le n \le N.$$
3. **Inner weighting (state update):** For each state particle $\textbf{x}_t^{(n,m)}$, compute the weight $w_t^{(n,m)} \propto p\bigl (\textbf{Y}_t \mid \textbf{x}_t^{(n,m)}\bigr ),$
4. **Outer weighting (parameter update):** For each candidate $\bar{\boldsymbol{\theta }}^{(m)}_t$, approximate its marginal likelihood by averaging the inner weights:
  $$u_t\bigl (\bar{\boldsymbol{\theta }}^{(m)}_t\bigr ) \approx \frac{1}{N}\sum _{n=1}^{N} p\bigl (\textbf{Y}_t \mid \textbf{x}_t^{(n,m)}\bigr ), \qquad v_t^{(m)} \propto u_t\bigl (\bar{\boldsymbol{\theta }}^{(m)}_t\bigr ).$$
5. **Parameter resampling:** Resample the parameter particles (and their associated state particles) according to $v_t^{(m)}$ to yield the updated set $\{\boldsymbol{\theta }_t^{(m)},\, \{\textbf{x}_t^{(n,m)}\}_{n=1}^{N}\}_{m=1}^{M}$.
  This collection approximates the joint posterior distribution of $\textbf{X}_t$ and $\theta$.

However, NPF algorithm tracks the states and parameters $(\textbf{X}_t,\boldsymbol{\theta })$ using observation only up till time *t*. The noise abduction step requires the full set of observations (i.e., up to time *T*). Therefore, once the forward filtering is complete, we apply a backward smoothing pass to refine the state and parameter estimates by incorporating information from future observations $\textbf{Y}_{t:T}$.

**Inferring the exogenous noise**

Once the hidden states are estimated, the abducted noise variables are computed using the structural equation in (2). Specifically, with every state and parameter particle $(\textbf{x}_{t}^{(n, m)}, \boldsymbol{\theta }^{(m)})$ the corresponding noise $\boldsymbol{\mu }_{t}^{(n,m)}$ is calculated as the residual of the following form:

4 $$\begin{aligned} \boldsymbol{\mu }_{t}^{(n,m)} = \textbf{x}_{t}^{(n, m)} - F\left( \textbf{x}_{t-1}^{(n, m)}, \boldsymbol{\theta }^{(m)}\right) . \end{aligned}$$

Then the abducted noise ${\textbf{U}_t^{\text {cf}}}$ can be approximated with a normal distribution with mean $\boldsymbol{\mu }_t$ and variance $\boldsymbol{\sigma }_t.$ The mean $\boldsymbol{\mu }_t$ is calculated as the weighted average of the residuals $\boldsymbol{\mu }_{t}^{(n, m)}$ at each time *t*. Concretely, ${\textbf{U}_t^{\text {cf}}}$ is sampled from $\mathcal {N}(\boldsymbol{\mu }_t, \boldsymbol{\sigma }_t)$, with

$$\begin{aligned} \boldsymbol{\mu }_t = \sum _{n, m = 1}^{N, M} \tilde{w}_t^{(m,n)} \left( \textbf{x}_{t}^{(n, m)} - F(\textbf{x}_{t-1}^{(n, m)}, \boldsymbol{\theta }^{(m)})\right) \quad \text {and} \quad \boldsymbol{\sigma }_t = \sum _{n, m = 1}^{N, M} \tilde{w}_t^{(m,n)}\left( \boldsymbol{\mu }_t^{(n,m)} - \boldsymbol{\mu }_t\right) ^2, \end{aligned}$$

where $\tilde{w}_t^{(m,n)}$ are the normalized smoothed weights returned by the backward smoothing layer. Here, $\boldsymbol{\sigma }_t$ is the weighted variance.

### Generating counterfactual trajectories

To generate the counterfactual sequence, we first define the counterfactual Structural Causal Model (CF-SCM), which -given the abducted noise $\textbf{U}_t^{\text {cf}}$- is specified according to the type of intervention. In this work, we mainly focus on interventions on the initial conditions: how the sequence of hidden states would evolve if the initial state had been different. Specifically, if the initial state was slightly perturbed with an insignificant value $\delta,$ ($\textbf{X}_0^{cf} = \textbf{X}_0 + \delta \textbf{e}_j$), how would the rest of the sequence propagate $\textbf{X}_{1:t}^{cf}$? This is particularly relevant in the context of chaotic systems, as a slight change in initial conditions would lead to vastly different sequences.

5 $$\begin{aligned} {\left\{ \begin{array}{ll} \textbf{X}^{\text {cf}}_{t} & := F_{t}\left( \textbf{X}^{\text {cf}}_{t-1}, \tilde{\boldsymbol{\theta }} \right) + \textbf{U}_t^{\text {cf}}, \quad \textbf{U}_t^{\text {cf}} \sim \mathcal {N}\left( \ \boldsymbol{\mu }_t, \boldsymbol{\sigma }_t\right) .\\ \textbf{X}_0^{cf} & = \textbf{X}_0 + \delta \textbf{e}_j, \end{array}\right. } \end{aligned}$$

with $\delta >0$ is a small perturbation and $\textbf{e}_j$ is *j*-th unit vector. Note that if the governing parameters in the CF-SCM reflect the true underlying dynamics, $\tilde{\boldsymbol{\theta }} = \boldsymbol{\theta }_{true}$, the counterfactual trajectory should closely resemble the deterministic counterfactual projection, especially when the system is endowed with a structure a priori. However, if the parameter estimates are slightly inaccurate, $\tilde{\boldsymbol{\theta }} = \hat{\boldsymbol{\theta }}$, the counterfactual predictions can become unreliable. This unreliability arises because chaotic systems exhibit unpredictable behavior for certain parameters while being completely predictable for others. To this end, we consider three options for $\tilde{\boldsymbol{\theta }}$ in Eq. (5):

- $\tilde{\boldsymbol{\theta }}$ is set to the *true* parameter values ($\tilde{\boldsymbol{\theta }} = \boldsymbol{\theta }_{true}$). In this configuration of the CF-SCM, there is no uncertainty in the underlying dynamics of the counterfactual scenario. The reliability of CF trajectories is influenced solely by the estimated posterior distribution of the noise.
- $\tilde{\boldsymbol{\theta }}$ is set to the *estimated* parameter ($\tilde{\boldsymbol{\theta }} = \hat{\boldsymbol{\theta }}$), here $\hat{\boldsymbol{\theta }}$ is obtained using the filtering-smoothing algorithm described in section “Background”. This setting allows us to assess counterfactual trajectories when a slight inaccuracy is introduced into the underlying dynamics of the system.
- $\tilde{\boldsymbol{\theta }}$ is drawn from the full posterior distribution of the parameters $\mathcal {N}(\hat{\boldsymbol{\theta }}, \boldsymbol{\sigma }_{\boldsymbol{\theta }})$. This posterior distribution captures the uncertainty around the parameter estimates, reflecting the range of plausible values given the full length of observations and model assumptions. This approach assesses the reliability of the counterfactual reasoning while accounting for parameter uncertainty, simulating a more realistic scenario where the true parameter values are not known precisely.

## Simulated experiments

**Fig. 2:**
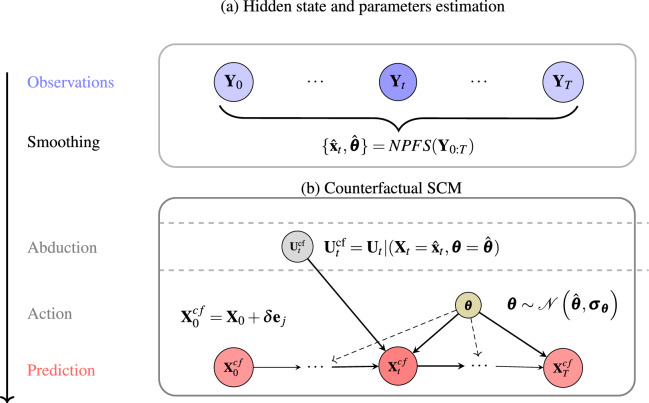
Illustration of the proposed methodology to generate counterfactuals. Panel (**a**) shows the estimation of states and parameters, using the filtering/smoothing (NPFS) method, given a sequence of observations $\textbf{Y}_{0:T}.$ Panel (**b**) CF-SCM: after abducting the noise $\textbf{U}_t^{\text {cf}}$, an intervention is applied to the initial state $\textbf{X}_{0}$, resulting in a counterfactual trajectory $\textbf{X}_{0:T}^{\text {cf}}$.

This section presents simulation results that illustrate the sensitivity of counterfactual reasoning in dynamical systems. The simulations are intentionally simple, designed to approximate real-world scenarios under controlled conditions, and are performed on systems that are nearly at a toy level. Specifically, we investigate how common assumptions in the mathematical modeling of real-world systems, such as noisy observations, model uncertainty, and chaotic dynamics, affect the quality and reliability of counterfactual reasoning within these systems. The flow of our approach is summarized in Fig. 2. We generate a noisy trajectory by simulating a dynamical ’ground truth’ model from the literature with known parameters. Next, we apply the filtering/smoothing technique from section “Methodology” to estimate both hidden states and system parameters. Using these estimates, we approximate the posterior noise distribution. The CF-SCM is then constructed by incorporating the abducted noise and performing an intervention at the initial state level. We generate multiple counterfactual trajectories by sampling from the estimated posterior noise, with the parameter node $\tilde{\boldsymbol{\theta }}$ in the CF-SCM following the three options described in the previous subsection.

We evaluated counterfactual reliability on several dynamical systems, including the Lorenz, Rössler, and Logistic Growth models, using an NPF for state and parameter estimation. The specifics of the models, initial conditions, parameter priors, and evaluation metrics are thoroughly described in the Supplementary Information.

To evaluate the reliability of counterfactual sequences, we compare the estimated counterfactual sequences, $\textbf{X}^{\text {cf}_i}_{1:T}$, and the deterministic counterfactual sequence, $\textbf{X}_{1:T}^{\text {cf}}$. This comparison is conducted through one- and two-dimensional plots, along with a line plot of the Root Mean Squared Error across time (RMSE$_t$; see Supplementary Information). Here, $\textbf{X}^{\text {cf}}_{1:T}$ denotes the deterministic counterfactual trajectory, obtained by propagating the system forward from a counterfactual initial condition, $\textbf{X}_0^{\text {cf}}$, using noise-free dynamics. Although this setting is idealized, it provides a controlled reference against which the generated counterfactuals can be assessed, highlighting how closely they approximate the true counterfactual trajectory.

### Simulation results

In this section, we present the results of our simulation experiments based on the counterfactual sequence generation process described in the previous section. Specifically, the parameter $\tilde{\boldsymbol{\theta }}$ in the CF-SCM, can be set to the true parameters $\boldsymbol{\theta }_{true}$, the point estimate $\hat{\boldsymbol{\theta }}$, or drawn from the approximated posterior parameters $\mathcal {N}(\hat{\boldsymbol{\theta }}, \boldsymbol{\sigma }_{\boldsymbol{\theta }}).$ Observational noise and process noise are sampled from a normal distribution with mean $\textbf{0}$ and variances $\sigma _{\textbf{W}}\textbf{I}$ and $\sigma _{\textbf{U}}\textbf{I}$, respectively, with $\sigma _{\textbf{W}}$ and $\sigma _{\textbf{U}}$ taking values in $\left\{ (0.01,4), (0.01,9), (1,2), (4,1)\right\}$. The initial conditions for the Lorenz, Rössler and logistic growth systems are set to (1, 1, 1) and (1, 1, 0), and 10 respectively. The counterfactual initial conditions are defined for Lorenz and Rössler systems as $\textbf{X}^{\text {cf}}_0 = \textbf{X}_0 + 10^{-4}\textbf{e}_1$, where $\textbf{e}_1$ represents a unit perturbation along the first coordinate. For logistic growth, the initial condition is set to $X^{\text {cf}} = X_0 + 10$.

**Fig. 3:**
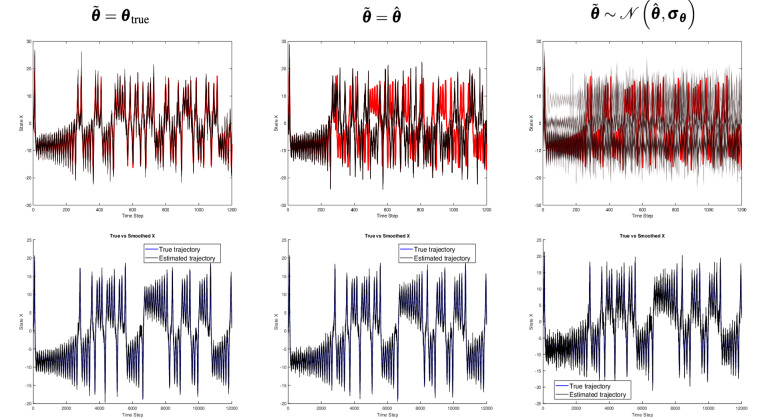
Top row: Deterministic counterfactual trajectory (in red) compared to the generated counterfactual trajectories (in black) for the Lorenz system under three parameter assumptions: $\tilde{\boldsymbol{\theta }} = {\boldsymbol{\theta }}_{true}$, $\tilde{\boldsymbol{\theta }} = \hat{\boldsymbol{\theta }}$, and $\tilde{\boldsymbol{\theta }} \sim \mathcal {N}(\hat{\boldsymbol{\theta }}, \boldsymbol{\sigma }_{\boldsymbol{\theta }})$. Bottom row: Observed sequence (black) alongside the estimated factual hidden sequence (blue), illustrating how even an accurate factual estimation may fail to produce reliable counterfactual trajectories once initial conditions are altered or parameter uncertainty is introduced.

**Fig. 4:**
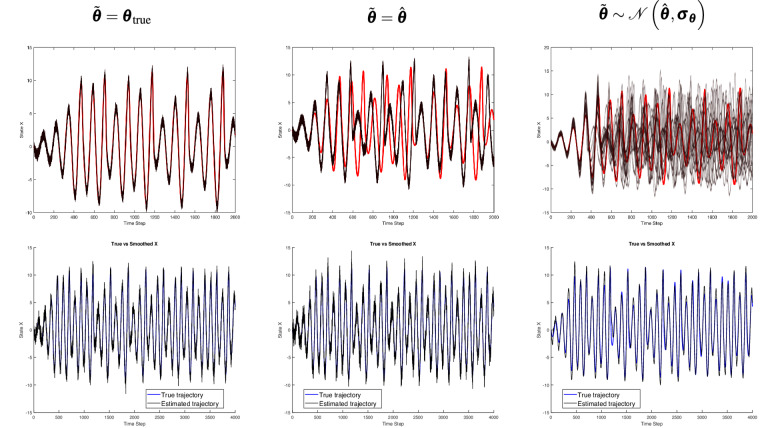
Top row: Deterministic counterfactual trajectory (in red) compared to the generated counterfactual trajectories (in black) for the Rössler system under three parameter assumptions: $\tilde{\boldsymbol{\theta }} = {\boldsymbol{\theta }}_{true}$, $\tilde{\boldsymbol{\theta }} = \hat{\boldsymbol{\theta }}$, and $\tilde{\boldsymbol{\theta }} \sim \mathcal {N}(\hat{\boldsymbol{\theta }}, \boldsymbol{\sigma }_{\boldsymbol{\theta }})$. Bottom row: Observed sequence (black) alongside the estimated factual hidden sequence (blue), illustrating how even an accurate factual estimation may fail to produce reliable counterfactual trajectories once initial conditions are altered or parameter uncertainty is introduced.

**Fig. 5:**
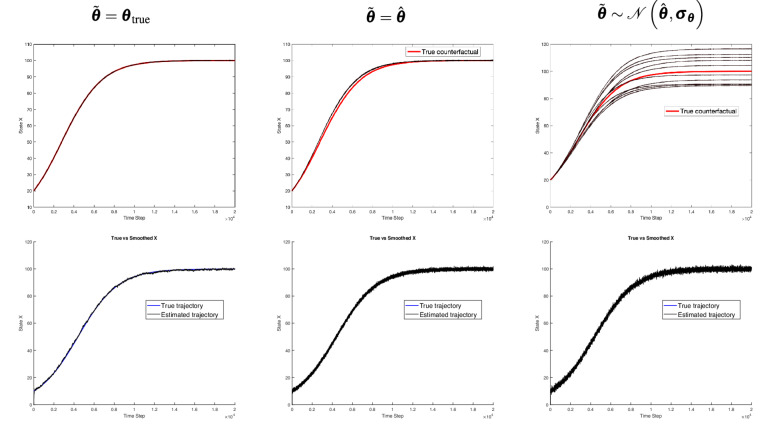
Top row: Deterministic counterfactual trajectory (in red) compared to the generated counterfactual trajectories (in black) for the logistic growth system under three parameter assumptions: $\tilde{\boldsymbol{\theta }} = {\boldsymbol{\theta }}_{true}$, $\tilde{\boldsymbol{\theta }} = \hat{\boldsymbol{\theta }}$, and $\tilde{\boldsymbol{\theta }} \sim \mathcal {N}(\hat{\boldsymbol{\theta }}, \boldsymbol{\sigma }_{\boldsymbol{\theta }})$. Bottom row: Observed sequence (black) alongside the estimated factual hidden sequence (blue).

Figures 3,  4, and  5 represent results for Lorenz, Rössler and Logistic growth systems. The second row displays one-dimensional line plots of the estimated factual trajectory from the observational trajectory, while the first row displays one-dimensional line plots of the generated and deterministic counterfactuals. The first, second and third columns show how system parameters are incorporated in the CF-SCM namely, $\tilde{\boldsymbol{\theta }} = {\boldsymbol{\theta }}_{\text {true}}$, $\tilde{\boldsymbol{\theta }} = \hat{\boldsymbol{\theta }}$ and $\tilde{\boldsymbol{\theta }} \sim \mathcal {N}(\hat{\boldsymbol{\theta }}, \boldsymbol{\sigma }_{\boldsymbol{\theta }})$, respectively.

**Fig. 6:**
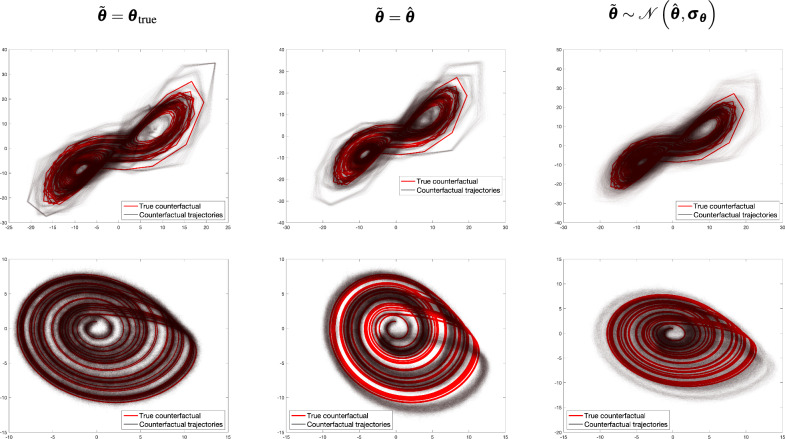
2D plot of generated counterfactual trajectories in black and the deterministic counterfactual trajectory in red for Lorenz (first row) and Rössler (second row) systems.

**Fig. 7:**
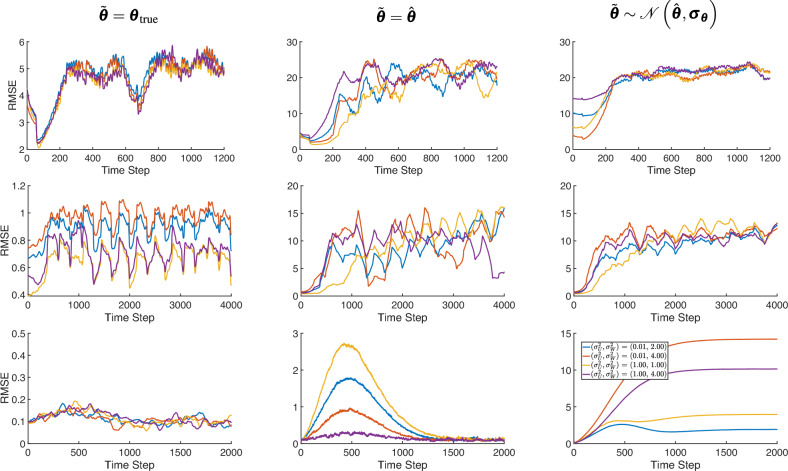
RMSE$_t$ of counterfactual trajectories generated for four different noise values, corresponding to the Lorenz (first row), Rössler (second row), and Logistic growth (third row) systems.

Figure 6 displays the two-dimensional plot of the deterministic and generated counterfactual trajectories of Lorenz and Rössler in the first and second row, respectively. The first, second and third columns show how system parameters are incorporated in the CF-SCM namely, $\tilde{\boldsymbol{\theta }} = {\boldsymbol{\theta }}_{\text {true}}$, $\tilde{\boldsymbol{\theta }} = \hat{\boldsymbol{\theta }}$ and $\tilde{\boldsymbol{\theta }} \sim \mathcal {N}(\hat{\boldsymbol{\theta }}, \boldsymbol{\sigma }_{\boldsymbol{\theta }})$, respectively. Figure 7 represents the line plot of RMSE$_t$ error as time *t* progresses. As outlined in the Supplementary Information, the error is calculated based on the generated versus deterministic counterfactuals. Note that RMSE reported is smoothed with a window size of 200 discrete time steps. In the Lorenz and Rössler systems, both known for their chaotic dynamics, we observed a pronounced sensitivity to initial conditions, as shown in the one-dimensional line plots in Figs.  3 and  4. With parameters set to the true values $\tilde{\boldsymbol{\theta }} = \boldsymbol{\theta }_{\text {true}}$, the counterfactual trajectories closely align with the deterministic counterfactual in the initial time steps but soon diverge substantially, which is more pronounced in the two-dimensional representation in Fig. 6. This behavior underscores the “butterfly effect,” where small deviations lead to vastly different outcomes over time, even if the underlying parameters are considered to be known a priori.

When parameters are set to point estimates $\tilde{\boldsymbol{\theta }} = \hat{\boldsymbol{\theta }}$ or sampled from an approximated posterior distribution $\mathcal {N}(\hat{\boldsymbol{\theta }}, \boldsymbol{\sigma }_{\boldsymbol{\theta }})$, Figs. 3,  4 (first line), and  6 reveal a substantial increase in trajectory divergence. In Figure 7, the RMSE over time indicates a significant and persistent error for chaotic systems, particularly in the second and third columns, where parameter uncertainties are introduced. This behavior is indeed attributed to sensitive dependence on initial conditions, a key attribute in chaotic systems. However, the second line of Figs. 3 and 4 explicitly shows the corresponding factual trajectories estimated from observational sequences. This reinforces that, while parameter inference techniques can effectively approximate system dynamics, even slight inaccuracies in parameter estimates can drastically alter the reliability of counterfactual sequences in chaotic systems. Figure 7 also illustrates that as noise levels increase, the RMSE remains consistently high throughout the trajectory, especially in the Lorenz and Rössler systems. This high RMSE reflects the compounding effect of noise in a chaotic system, where small errors in state estimation quickly amplify, thereby reducing counterfactual reliability. Here, the logistic growth system serves as a baseline for comparison with the chaotic Lorenz and Rössler models, precisely because it is not inherently chaotic. The counterfactual trajectories in the first row of Fig. 5 and the recorded errors in Fig. 7 show that when the parameters are known or shifted ever so slightly, the counterfactual sequences remain aligned with the deterministic trajectory. In the third row, uncertainty and noise are evident in the spread of counterfactuals, yet the system moves into a stable state by around time step (1000), keeping deviations within acceptable bounds. However, we stress that these experiments rely on strong assumptions of additive Gaussian noise and perfect structural knowledge of the ODE, made intentionally to isolate and highlight how chaos interacts with noise and uncertainty. While non-chaotic systems may appear to yield more robust counterfactual predictions under these conditions, real-world settings that lack such idealized knowledge–and that may involve additional confounders or more complex noise processes–remain challenging for counterfactual reasoning.

Figure 7 further explores the sensitivity of counterfactual reliability by varying both process and observation noise across four different settings, while also contrasting three parameter specifications (ground truth parameters, point estimates, and drawn from posterior distribution of parameters obtained by filtering/smoothing). The Lorenz and Rössler systems (first and second row) show rapid error growth, especially when posterior-sampled parameters are used (third column). Crucially, when true parameters or point estimates are used (first and second column), we observe a “sweet spot” window: after parameters are well learned (evidenced by a dip in RMSE), counterfactuals remain briefly reliable before noise accumulation and chaos dominate. Additional figures in the “Supplementary Information” section provide additional results for different process and observational noise values.

Notably, the Lorenz system’s characteristic sensitivity appears around time step 500 in Fig. 3, even when parameters are set to their true values, because slight differences in initial conditions rapidly accumulate. However, once parameter uncertainty is introduced, these minor shifts in parameter space exacerbate the system’s natural divergence, causing counterfactual trajectories to deviate significantly from the deterministic baseline. This effect emerges even earlier, near time steps 300 and 10 in the second and third columns of Fig.  3, underscoring how parameter uncertainty can trigger divergence well before the 500-step mark that would otherwise be observed under true parameter settings.

**Table 1:** Dynamical systems considered in the experiments, along with their corresponding ground truth parameters, prior distributions, and chaotic behavior regions.

System	Ground truth $\boldsymbol{\theta }$	Prior distribution (Uniform)	Chaotic region
Lorenz system	$\left( \sigma , \rho , \beta \right) = \left( 10, 28,8/3 \right)$	$\sigma \sim U(5, 15)$ $\rho \sim U(20, 35)$ $\beta \sim U(2, 4)$	$\rho \gtrsim 24.74$
Rössler system	$\left( a,b,c \right) = \left( 0.2, 0.2, 5.7\right)$	$a \sim U(0.1, 0.3)$ $b \sim U(0.1, 0.3)$ $c \sim U(4, 7)$	$c \gtrsim 5.7$
Logistic growth	$\left( r, K \right) = \left( 3.9, 1\right)$	$r \sim U(2, 4)$ $K \sim U(0.8, 1.2)$	N/A

## Discussion, conclusions and future work

We use chaotic systems like Lorenz and Rössler models precisely to illustrate the fragility of counterfactual reasoning in dynamic settings where uncertainty, hidden states, and chaos are involved. In many real-world scenarios, the true states of systems are hidden, observations are noisy, and system parameters are uncertain. One might not even be aware that a system exhibits chaotic behavior. Therefore, we focus on using chaotic dynamical systems even under strong assumptions such as (1) known structure, (2) access to the full observation sequence, and (3) controlled prior distributions of the parameters. When counterfactual reasoning is applied even in such controlled contexts, the inherent unpredictability and sensitivity to initial conditions in chaotic systems can lead to significant deviations in the outcomes of counterfactual sequences, occurring well before any divergences appear in the predicted factual sequences. This demonstrates that counterfactual reasoning can be particularly fragile in the presence of chaos and uncertainty, highlighting the need for caution when applying these methods to complex dynamical systems. All models, including Structural Causal Models (SCMs), are approximations of reality. Computing counterfactuals relies on SCMs. A key insight is that while an SCM may suffice for prediction or causal inference, it may not be reliable for counterfactual estimation. Common approximations–like parameter uncertainties, complex underlying dynamics, or simplifying abstractions–can undermine the reliability of counterfactual estimations.

Our results indicate that there are fundamental theoretical and practical limitations to computing counterfactuals, which require further refinement in future work. These limitations could be particularly relevant in fields such as medicine, where counterfactual insights hold great promise for personalized treatment. Additionally, our findings highlight the difficulty of formalizing certain aspects of human counterfactual reasoning within SCMs.

Three open problems emerge from our study: quantifying the impact of chaos on posterior densities by extending the nested particle filtering framework, identifying whether coarser abstractions can stabilize counterfactual reliability when fine-grained trajectories diverge, and determining the temporal windows within which reasoning counterfactually remains tractable. That is, temporal upper and lower bounds where interventions are both informative and timely, rather than premature (dominated by model uncertainty) or too late (rendered meaningless by compounding noise).

Returning to our initial example of Alice passing her final exam, it might be straightforward to compute specific counterfactuals that would make the event impossible (e.g., Alice opening a bakery instead of attending college). However, we currently lack the formalism to adequately study such abstract forms of reasoning and their likelihoods. We hope future work will shed more light on this important aspect of causal research.

## Supplementary Information

Supplementary Information.

## Data Availability

All datasets used in this study are synthetic. They were generated by numerically integrating the ordinary differential equations described in Section “Experiments”, with the corresponding parameters explicitly listed in Table 1.
