# Supplementary material for: Chaotic and complex dynamics expose the limits of counterfactual reasoning
Source: Sci Rep. 2026 Jun 2;16:16978. doi: 10.1038/s41598-026-52349-2 (PMC13230520; doi:10.1038/s41598-026-52349-2)
Supplement: Supplementary file 1 — Supplementary Information. [file 41598_2026_52349_MOESM1_ESM.pdf]

Chaotic and Complex Dynamics Expose the Limits of Counterfactual Reasoning

Yahya Aalaila[1,3,*], **Gerrit Großmann**[1], **Sumantrak Mukherjee**[1], **Jonas Wahl**[2], **and Sebastian Vollmer**[1]

[1]German Research Center for Artificial Intelligence (DFKI), Data Science and its Applications Research Group, Kaiserslautern, Germany
[2]German Research Center for Artificial Intelligence (DFKI), Research Department Neuro-Mechanistic Modeling, Saarbrücken, Germany
[3]Mohammed VI Polytechnic University, UM6P College of Computing, Benguerir, Morocco
[*]yahya.aalaila@dfki.de

ABSTRACT

Counterfactual reasoning, a cornerstone of human cognition and decision-making, is often seen as the "holy grail" of causal learning, with applications ranging from interpreting machine learning models to promoting algorithmic fairness. While counterfactual reasoning has been extensively studied in contexts with clearly defined static causal models, many real-world scenarios reside in dynamic settings often involving model and parameter uncertainty, observational noise, and chaotic behavior. The reliability of counterfactual analysis in such settings remains largely unexplored. In this work, we investigate the limitations of counterfactual reasoning in dynamic settings. We specifically focus on counterfactual sequence estimation and demonstrate empirically that even modest levels of model uncertainty or observational noise can lead to dramatic deviations between predicted and true counterfactual trajectories. Our findings urge caution when applying counterfactual reasoning in dynamical systems, particularly those that may exhibit complex, chaotic behavior, and highlight fundamental limitations in answering certain counterfactual queries reliably.

Appendix

Running Example

The Lorenz System as an ODE

Consider the Lorenz system, a set of three coupled, nonlinear differential equations originally developed to model atmospheric convection. The system is known for its chaotic behavior under certain parameter values and initial conditions. Let $\boldsymbol{X}(t) = (X_1(t), X_2(t), X_3(t))$ denote the state variables at time t, representing the convective fluid velocity, horizontal temperature variation, and vertical temperature variation, respectively. Let $h_{\boldsymbol{\theta}}(\boldsymbol{X}(t)) = \big(h_{1,\boldsymbol{\theta}}(\boldsymbol{X}(t)), h_{2,\boldsymbol{\theta}}(\boldsymbol{X}(t)), h_{3,\boldsymbol{\theta}}(\boldsymbol{X}(t))\big)$. The system is described by the following ODEs:

$$\begin{cases} \frac{d}{dt}X_1(t) = h_{1,\boldsymbol{\theta}}\big(\boldsymbol{X}(t)\big) = \sigma\left(X_2(t) - X_1(t)\right), \\ \frac{d}{dt}X_2(t) = h_{2,\boldsymbol{\theta}}\big(\boldsymbol{X}(t)\big) = X_1(t)\left(\rho - X_3(t)\right) - X_2(t), \\ \frac{d}{dt}X_3(t) = h_{3,\boldsymbol{\theta}}\big(\boldsymbol{X}(t)\big) = X_1(t)X_2(t) - \beta X_3(t), \\ (X_1(0), X_2(0), X_3(0)) = \left(X_1^0, X_2^0, X_3^0\right), \end{cases} \tag{1}$$

where $\boldsymbol{\theta} = (\sigma, \rho, \beta)$, with $\sigma, \rho, \beta > 0$ being constants.

This system models the dynamics of convection rolls in the atmosphere and is notable for its chaotic solutions under certain conditions. The functions $h_{1,\boldsymbol{\theta}}$, $h_{2,\boldsymbol{\theta}}$, and $h_{3,\boldsymbol{\theta}}$ represent the rate of change for each state variable.

Lorenz system and SSM

The forward operator $F(\mathbf{X}_{t-1}, \boldsymbol{\theta})$ represents the deterministic dynamics of the Lorenz system, modeling the interactions among the convective fluid velocity and the horizontal and vertical temperature variations. We can define it the fourth-order Runge-Kutta, as:

$$F(\mathbf{X}_{t-1}, \boldsymbol{\theta}) = \mathbf{X}_{t-1} + \frac{\Delta}{6}\left(k_{1,\boldsymbol{\theta}} + 2k_{2,\boldsymbol{\theta}} + 2k_{3,\boldsymbol{\theta}} + k_{4,\boldsymbol{\theta}}\right). \tag{2}$$

where $\Delta \in \mathbb{R}_{>0}$ is the size of the discrete time step and $\boldsymbol{\theta} = (\sigma, \rho, \beta)$. Here, $k_{1,\boldsymbol{\theta}}$, $k_{2,\boldsymbol{\theta}}$, $k_{3,\boldsymbol{\theta}}$ and $k_{4,\boldsymbol{\theta}}$ are defined as

$$k_{1,\boldsymbol{\theta}} = \mathbf{h}_{\boldsymbol{\theta}}(\mathbf{X}_{t-1}), \tag{3}$$

$$k_{2,\boldsymbol{\theta}} = \mathbf{h}_{\boldsymbol{\theta}}\left(\mathbf{X}_{t-1} + \frac{\Delta}{2}k_{1,\boldsymbol{\theta}}\right), \tag{4}$$

$$k_{3,\boldsymbol{\theta}} = \mathbf{h}_{\boldsymbol{\theta}}\left(\mathbf{X}_{t-1} + \frac{\Delta}{2}k_{2,\boldsymbol{\theta}}\right), \tag{5}$$

$$k_{4,\boldsymbol{\theta}} = \mathbf{h}_{\boldsymbol{\theta}}\left(\mathbf{X}_{t-1} + \Delta k_{3,\boldsymbol{\theta}}\right). \tag{6}$$

Lorenz system as an SCM

We aim to represent the State Space Model describing the Lorenz system as an SCM. To achieve this, we define the following variables:

- $V_t^{X_{1,t}} \in \mathbb{R}$, $V_t^{X_{2,t}} \in \mathbb{R}$, and $V_t^{X_{3,t}} \in \mathbb{R}$ denote the true state variables of the Lorenz system at time at time $t \in \{0, \ldots, T\}$.
- $V_t^{Y_{1,t}} \in \mathbb{R}$, $V_t^{Y_{2,t}} \in \mathbb{R}$, and $V_t^{Y_{3,t}} \in \mathbb{R}$ represent the corresponding noisy observations at time t.

For each time point t, we introduce two noise variables. $\mathbf{U}_t \in \mathbb{R}^3$ accounts for process noise and $\mathbf{W}_t \in \mathbb{R}^3$ accounts for observational noise. Variables at the initial time $t = 0$ have no parent dependencies. For $t > 0$, the dependencies are structured as follows:

- Each observational variable $V_t^{Y_{i,t}}$ is influenced by the true state variable $V_t^{X_{i,t}}$ and the observational noise $\mathbf{W}_t$.
- Each true state variable $V_t^{X_{i,t}}$ depends on the previous true state variables $V_t^{X_{1,t}}$, $V_t^{X_{2,t}}$, and $V_t^{X_{3,t}}$, as well as the process noise $\mathbf{U}_t$.

The functional relationships are defined by the RK4 functions defined in equation (2). To incorporate the parameter vector $\boldsymbol{\theta} = (\sigma, \rho, \beta)$ into the model, we introduce an additional node representing $\boldsymbol{\theta}$.

Experiments

Experimental Set-up

We use different dynamical systems described by an ordinary differential equation. Table **??** summarizes these systems with the corresponding setup. We use the state space model to describe the evolution of system's state over time. The forward deterministic pass $F(\mathbf{X}_{t-1}, \boldsymbol{\theta})$ in the state equation is set to the RK4 approximation method.

True states and noisy observations of the system are generated using a user-defined initial condition $\mathbf{X}_0$ and true parameters $\boldsymbol{\theta}_{true}$. Based on the generated observations, we estimate the hidden states and system parameters using a forward-nested filter followed by a backward-smoothing pass. We generate counterfactual trajectories based on interventions on the initial conditions. We abduct the noise posterior based on the estimated states and parameters. The sequences of counterfactual hidden states are generated based on equation (**??**).

The number of particles in the filtering process is set to $N = M = 200$. Initial particles $\theta^{(m)}$ are generated from a prior distribution π_0 that depends on the dynamical system. Process noise $\mathbf{U}_t$ is sampled from normal distribution with mean $\mathbf{0}$ and variance $a\mathbf{I}$. Observational noise $\mathbf{V}_t$ is sampled from normal distribution with mean $\mathbf{0}$ and variance $b\mathbf{I}$. The step size Δ in RK4 is set to 5×10^{-2}. All experiments were conducted using Matlab R2024b.

Dynamical systems Table **??** details the dynamical systems considered in the experiments, mainly Lorenz, Rössler, and Logistic Growth systems. For each system, we specify the initial conditions, the "ground truth" parameters, the prior distributions used in the particle filter (PF), and the regions of chaotic behavior.

The Lorenz and Rössler systems are classic examples of chaotic dynamical systems, both characterized by a set of three coupled, nonlinear differential equations. The Lorenz system, originally developed to model atmospheric convection, is famous for its chaotic behavior, where small changes in initial conditions can lead to vastly different outcomes—an effect commonly referred to as the "butterfly effect." Similarly, the Rössler system was introduced as a simpler model of chaos and exhibits comparable sensitivity to initial conditions, where even slight perturbations can cause divergent trajectories over time. Both systems serve as important case studies in the field of chaos theory, providing insight into how chaotic dynamics manifest in different mathematical models.

We use the RK4 discrete-time approximation for both systems. Both systems are described by a three-dimensional state vector $\mathbf{X}_t = (X_{1,t}, X_{2,t}, X_{3,t})$, with parameters $\boldsymbol{\theta} = (\sigma, \rho, \beta) = (10, 28, 8/3)$ and $\boldsymbol{\theta} = (a, b, c) = (0.2, 0.2, 5.7)$. for Lorenz system. the initial particles are sampled from uniform prior distributions: $\sigma \sim \mathscr{U}(5, 20)$, $\rho \sim \mathscr{U}(15, 50)$, and $\beta \sim \mathscr{U}(1, 8)$. The initial

condition is set as $\mathbf{X}_0 = (1,1,1)$, and the counterfactual initial condition is defined as $\mathbf{X}_0^{\text{cf}} = \mathbf{X}_0 + 10^{-4}\mathbf{e}_1$. Similarly, for the Rössler system, the prior distributions for the parameters are given by $a \sim \mathscr{U}(0.1, 0.3)$, $b \sim \mathscr{U}(0.1, 0.3)$, and $c \sim \mathscr{U}(4, 8)$. The initial condition is $\mathbf{X}_0 = (1,1,0)$, and the corresponding counterfactual initial condition is $\mathbf{X}_0^{\text{cf}} = \mathbf{X}_0 + 10^{-4}\mathbf{e}_1$.

In contrast, the logistic growth model serves as a benchmark example of a non-chaotic system. It describes the evolution of a population over time, where growth is initially exponential but eventually stabilizes as the population approaches a carrying capacity. Unlike the Lorenz and Rössler systems, the logistic growth model does not exhibit sensitivity to initial conditions in the same way, making it a suitable reference for studying counterfactual reliability in the absence of chaotic dynamics. The logistic growth process is described by the discrete-time RK4 approximation of the logistic differential equation, with parameters $\boldsymbol{\theta} = (r, K) = (0.5, 100)$, where r is the intrinsic growth rate and K is the carrying capacity. The parameter priors are given by $r \sim \mathscr{U}(0,1)$ and $K \sim \mathscr{U}(85, 110)$. The initial condition is set to $X_0 = 10$, and the counterfactual initial condition is $X_0^{\text{cf}} = X_0 + 10$.

Evaluation metrics We calculate the root mean squared error (RMSE_t) over time to evaluate the accuracy of the sampled counterfactual trajectories compared to the projected counterfactual trajectory. This is done by computing the Euclidean distance at each time step in the d-dimensional phase space. Given d as the state dimension, T as the number of time steps, and N_{cf} as the number of counterfactual trajectories, the phase space distance between the deterministic counterfactual state $\mathbf{X}_t^{\text{cf}}$ and the i-th counterfactual state $\mathbf{X}_t^{\text{cf}_i}$ at time t is calculated as:

$$d(t,i) = \sqrt{\sum_{k=1}^{d} \left(\mathbf{X}_{k,t}^{\text{cf}_i} - \mathbf{X}_{k,t}^{\text{cf}}\right)^2}$$

where $\mathbf{X}_t^{\text{cf}}$ is the true counterfactual state computed using the deterministic ODE and $\mathbf{X}_t^{\text{cf}_i}$ the i-th counterfactual generated at time t, using equation (**??**). Here $\mathbf{X}_t^{\text{cf}} = [X_{1,t}^{\text{cf}}, X_{2,t}^{\text{cf}}, \ldots, X_{d,t}^{\text{cf}}]$ and $\mathbf{X}_t^{\text{cf}_i} = [X_{1,t}^{\text{cf}_i}, X_{2,t}^{\text{cf}_i}, \ldots, X_{d,t}^{\text{cf}_i}]$. The RMSE over all counterfactual trajectories at time t is then computed as:

$$\text{RMSE}_t = \sqrt{\frac{1}{N_{\text{cf}}} \sum_{i=1}^{N_{\text{cf}}} d(t,i)^2}$$

This provides a measure of how far, on average, the counterfactual trajectories deviate from the factual trajectory in the d-dimensional phase space, and it is calculated at each time step t.

Lorenz system

$$\begin{cases} \dfrac{dx_1}{dt} = \sigma(x_2 - x_1) \\ \dfrac{dx_2}{dt} = x_1(\rho - x_3) - x_2, \\ \dfrac{dx_3}{dt} = x_1 x_2 - \beta x_3 \end{cases} \qquad \boldsymbol{\theta} = \begin{pmatrix} \sigma \\ \rho \\ \beta \end{pmatrix} \tag{7}$$

Rössler system

$$\begin{cases} \dfrac{dx_1}{dt} = -x_2 - x_3 \\ \dfrac{dx_2}{dt} = x_1 + a x_2 \\ \dfrac{dx_3}{dt} = b + x_3(x_1 - c) \end{cases} \qquad \boldsymbol{\theta} = \begin{pmatrix} a \\ b \\ c \end{pmatrix} \tag{8}$$

Full Results

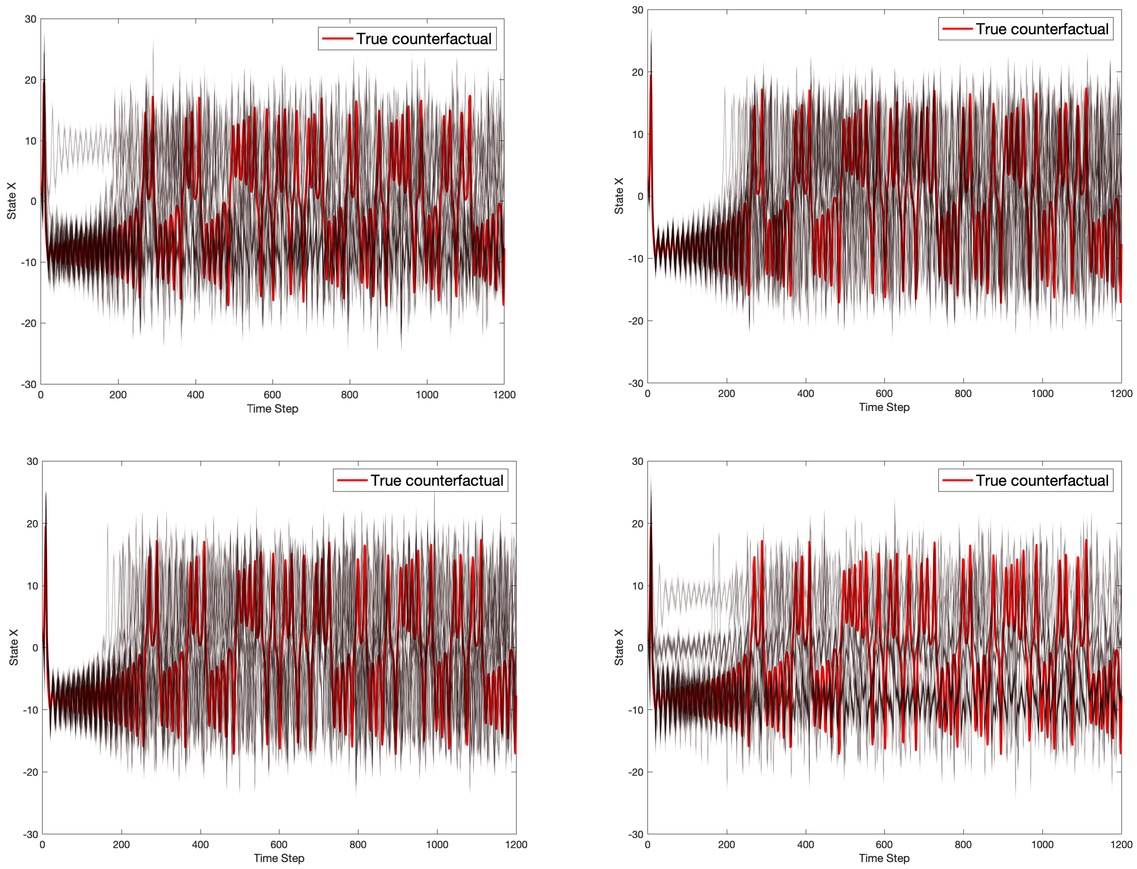

Figure 1. True counterfactual trajectory and generated counterfactual trajectories (in black) for Lorenz corresponding to $(\sigma_{\mathbf{U}}, \sigma_{\mathbf{W}}) = (4,1)$, $(1,2)$, $(0.01,4)$, and $(0.01,9)$ in the top left, top right, bottom left, and bottom right, respectively. The counterfactuals are generated by sampling values of the parameters from the posterior distribution.

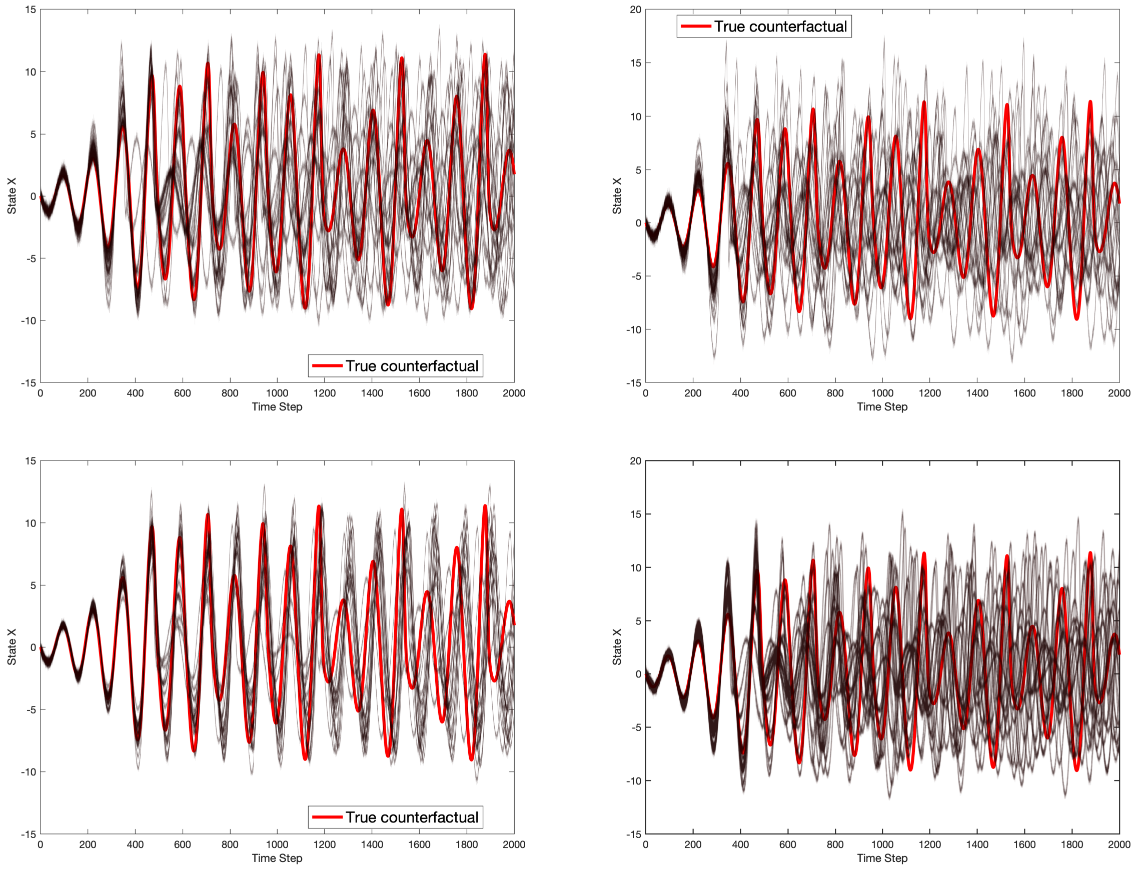

Figure 2. True counterfactual trajectory and generated counterfactual trajectories (in black) for Rössler, corresponding to $(\sigma_{\mathbf{U}}, \sigma_{\mathbf{W}}) = (4,1)$, $(1,2)$, $(0.01,4)$, and $(0.01,9)$ in the step left, top right, bottom left and bottom right, respectively. The counterfactuals are generated by sampling values of the parameters from the posterior distribution.
